# Double discharges in human soleus muscle

**DOI:** 10.3389/fnhum.2013.00843

**Published:** 2013-12-09

**Authors:** Maria Piotrkiewicz, Oğuz Sebik, Erdal Binboğa, Dariusz Młoźniak, Bożenna Kuraszkiewicz, Kemal S. Türker

**Affiliations:** ^1^Polish Academy of Sciences, Department of Engineering of Nervous and Muscular System, Nałęcz Institute of Biocybernetics and Biomedical Engineering, WarsawPoland; ^2^Laboratory of Neuromuscular Research, Koç University School of Medicine, IstanbulTurkey; ^3^Faculty of Medicine, Department of Biophysics, Ege University, IzmirTurkey

**Keywords:** soleus, double discharges, firing patterns, human motoneuron, delayed depolarization

## Abstract

Double discharges (doublets) were recorded from human soleus (SOL), where they have never been reported before. The data analyzed in this study were collected from 12 healthy volunteers. The subjects were recruited for other studies, concerning: (1) estimation of motoneurons’ (MNs) afterhyperpolarization (AHP) duration and (2) analysis of motor unit responses to nerve stimulation, and were not trained to voluntarily evoke doublets. The majority of intradoublet intervals fell into the commonly accepted range 2–20 ms. However, two SOL MNs from one presented exceptional doublets of intradoublet interval about 37 ms. This interval was virtually identical with the interval between second and third discharge in the few triplets recorded from another subject. It is hypothesized that triplets are generated by the delayed depolarization with the second narrow hump, which is the same as the hump responsible for exceptional doublets.

## Introduction

Motoneurons (MNs) during voluntary isometric contractions of a healthy human muscle discharge rhythmically with mean firing rates typically not exceeding 40/s. This type of discharge is controlled by afterhyperpolarization (AHP), which in MNs is longer than in many other types of nerve cell (Tripathy et al., 2012). However, some MNs are capable of firing double discharges (doublets) with short interspike intervals (ISIs) of a few milliseconds.

According to standard electromyographic terminology (AAEM, 2001), intradoublet ISIs should not exceed the range from 2 to 20 ms. However, detailed studies (Kudina, 1974; Halonen et al., 1977; Partanen and Lang, 1978; Bawa and Calancie, 1983; Kudina and Alexeeva, 1992b; Rowinska-Marcinska et al., 1999; Piotrkiewicz et al., 2008) have shown that the limits of this range can be sometimes exceeded and that each doublet is usually followed by prolonged ISI, which was considered a distinctive feature of doublets as early as in 1944 (Hoff and Grant). Moreover, in interval histograms of many doublet-firing MNs so-called “outsiders” can be seen, i.e., ISIs exceeding official intradoublet ISI range, but shorter than the lower limit of single ISI distribution.

Doublets are often recorded in non-physiological conditions, e.g., ischemia (Kugelberg, 1948) or neuromuscular disorders (Rowinska-Marcinska and Karwanska, 1994; Rowinska-Marcinska et al., 1999; Kostera-Pruszczyk et al., 2002; Piotrkiewicz et al., 2008) and therefore sometimes they have been considered to be a sign of MN dysfunction (Partanen and Lang, 1978). However, they can be also found in normal voluntary MN activity, although much more seldom (Denslow, 1948; Kudina, 1974; Andreassen and Rosenfalck, 1979; Bawa and Calancie, 1983). Single doublets are occasionally observed with MN recruitment, derecruitment or interspersed in rhythmic activity. Certain MNs are capable of firing series of intradoublet and interdoublet ISIs (repetitive doublets, Bawa and Calancie, 1983; Kudina and Churikova, 1990; Kirkwood and Munson, 1996; Kudina and Andreeva, 2010). The series may also be triggered through voluntary training (Bawa and Calancie, 1983; Kudina and Andreeva, 2010).

In animal studies, doublets were reported in cat (Calvin, 1974; Hoffer et al., 1987; Kirkwood and Munson, 1996) and rat (Gorassini et al., 2000) muscles. In the latter study, doublets were observed in fast hindlimb muscles (medial and lateral gastrocnemius and tibialis anterior), but not in the slow soleus (SOL). It should be mentioned, however, that the first evidence of doublets in animal experiment was obtained from the SOL of decerebrate cat (Eccles and Hoff, 1932; Hoff and Grant, 1944).

In human experiments, doublets were documented in flexor carpi radialis (Bawa and Calancie, 1983), flexor carpi ulnaris (Kudina and Churikova, 1990), extensor digitorum communis (Weber et al., 2009), palmaris longus (Bawa and Calancie, 1983), biceps brachii (Bawa and Calancie, 1983; Dengler et al., 1988; Piotrkiewicz et al., 2008), triceps brachii (Kudina and Andreeva, 2010), tibialis anterior (Andreassen and Rosenfalck, 1979, 1980), rectus femoris (Kudina, 1974) spinal extensors (Denslow, 1948), and trapezius (Denslow, 1948; Kudina and Alexeeva, 1992b; Kudina and Andreeva, 2010; Stephenson and Maluf, 2010). Trapezius and spinal extensors were reported to have the highest incidence of doublets (Denslow, 1948). To our knowledge, doublets have not been reported in human SOL.

The aim of this study was to document doublets observed in SOL muscle during long-lasting experiments that were designed for other purposes: investigation of AHP duration in human motoneurones (Piotrkiewicz et al., 2001) and study of responses to low-threshold stimulation of the tibial nerve (Binboğa et al., 2011).

## Methods

### Subjects

The data analyzed in this study were measured from previously recorded data sets (Piotrkiewicz et al., 2001; Binboğa et al., 2011). The 12 healthy volunteers, aged 21–58 (mean 38.3 years) were not trained to voluntarily evoke doublets and gave written informed consent to the experimental procedures that had gained Ethical Approval from the applicable institutional committees.

#### Experimental procedures

The detailed description of the experiments is given in previously published papers (Piotrkiewicz et al., 2001; Binboğa et al., 2011). Below, only the details relevant for the present study will be given.

##### Experiment 1



Data for the study of AHP duration were collected in the Kharkevich Institute for Information Transmission Problems, Russian Academy of Sciences, Moscow. During this investigation subjects were comfortably seated in an armchair and instructed to perform a series of isometric muscle contractions of various strengths keeping motor units (MUs) firing steadily with the help of auditory and visual feedback of the MU discharges. During the experiment, 5–21 constant-force electromyogram (EMG) fragments of 50–100 s duration were recorded from the SOL muscle. Between consecutive recordings, 3–4 min rest was provided. MU potentials were picked up by a bipolar needle electrode (DISA, 9013K0822), amplified by an electromyograph DISA A/S (Denmark, type 14 A 30) at 200–500 mV/cm with filters set at 20–10000 Hz, and stored on the magnetic tape for off-line analysis.

##### Experiment 2



Data for the study of MU responses to low-threshold electrical stimulation were obtained in the Centre for Brain Research at Ege University, Izmir, Turkey. During this investigation the subject lay prone on a physiotherapy table with right foot fixed to a force plate. The ankle angle was positioned at 90°. Subjects were instructed to gently plantar flex to recruit a single motor unit in SOL muscle. A single experiment lasted for 1–2.5 h. MU potentials were picked up from the SOL muscle by intramuscular custom made disposable bipolar wire electrodes, amplified (500x), filtered with a 200–10000 Hz band-pass filter, and stored by Cambridge Electronic Design (CED; UK) acquisition system for off-line analysis.

#### Data analysis

**Figure 1:**
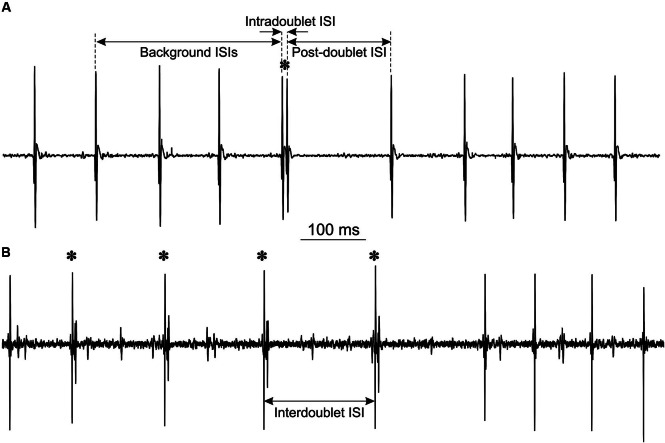
**Examples of MU potentials (doublets are marked by asterisks): (A)** single doublet, indicated are intervals, measured for double discharge analysis. Note the prolonged post-doublet ISI, which is one of the distinguishing features of “true” doublets. **(B)** Repetitive doublets.

All data were transferred to PCs by A/D converters with the sampling rates from 10 to 20 kHz, depending on the frequency content of the signal, so that there was no aliasing. Usually, potentials of a few MUs were recorded simultaneously in each experiment (Figure 1). Motor unit potential recordings from both experiments were decomposed off-line into single MU potential trains by an operator-computer interactive method using custom software described elsewhere (Mazurkiewicz and Piotrkiewicz, 2004) and subjected to common analysis described below.

**Figure 2:**
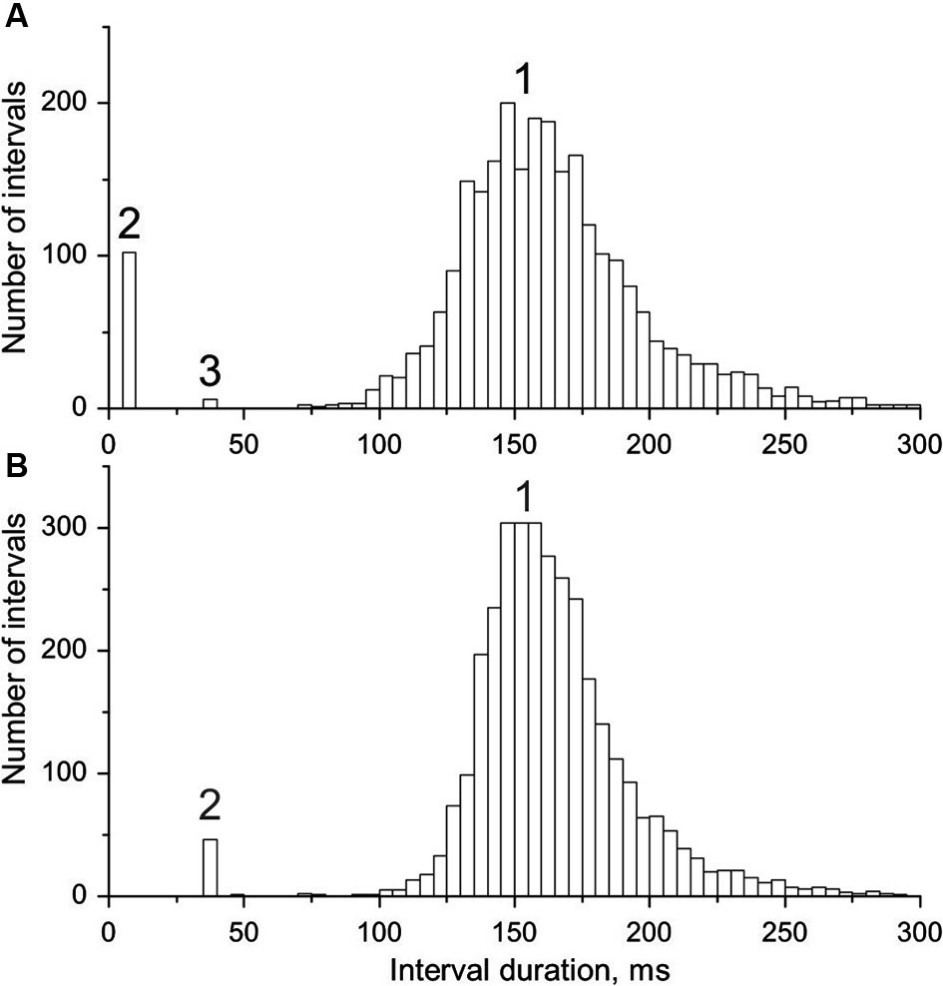
**Interval histograms of doublet-firing MUs. (A)** MU from group R. **(B)** MU from group E (see text for the explanation of the acronyms); 1, single, 2, double, 3, triple discharges. Note the coincidence of timing of triplets in A with doublets in B and some outsiders in both distributions.

 From the decomposed MU potential trains ISI histograms were constructed and those exhibiting bimodal distributions (see Figure 2) were searched for doublets. The search was based on the stereotyped firing pattern of doublets, which comprise “…two sequential firings of a motor unit action potential of the same form and nearly the same amplitude, occurring consistently in the same relationship to one another…” (AAEM, 2001) and are usually followed by a prolonged post-doublet ISI. The limits of intradoublet ISIs were determined from the histograms. Their upper limit sometimes exceeded 20 ms, specified by the official electromyographic terminology (AAEM, 2001).

The analysis included calculation of three ISIs for each doublet: mean background ISI from three consecutive intervals preceding the doublet, intradoublet ISI (between both doublet components) and post-doublet ISI (see Figure 1A for definitions). From these data the mean values and standard deviations of ISIs were calculated and histograms were plotted.

It was checked carefully if the doublets found in the data from the Experiment 2, were affected by the ongoing stimulation. This might have happened only if the second discharge had been synchronous with M- or H-response; however, such coincidences were not found.

## Results

Altogether, 169 MUs were identified in the SOL muscle. Sixteen MUs from this population were capable of firing doublets (incidence 9.5%). The range of MU firing rates was 3.5–15 imp/s. Our experiments, not specifically designed for the investigation of doublets in SOL, observed an unexpected number of doublets generated spontaneously during sustained contractions.

Characteristic features of doublet-firing MUs are collected in Table 1. MUs were classified into three groups. MUs from group S fired occasional *single doublets* interspersed in regular discharge (see the example in Figure 1A); these doublets constituted less than 0.5 % of all discharges. MUs from the group R fired both *single* and *repetitive doublets*, i.e., the series of doublet and post-doublet discharges; in this group, the doublets constituted more than 6% of all discharges (see the example in Figure 1B). Group E comprised two MUs from one subject, firing *exceptional doublets* (about 1% of all discharges). This group will be described separately with more detail.

In Figure 2 interval distributions of the two MUs with doublets are shown. Both have two separate maxima, one for regular discharges and the other for doublets. The histogram in Figure 2A has an additional maximum at around 36 ms, formed by six extremely long triplet intervals (35.9–36.5 ms; for more detailed description see the following section). Figure 2B presents the histogram of the MU with exceptional doublets. The duration of intradoublet ISI for this MU is within the limits 35.8–37.0 ms. Note striking similarity between both interval ranges (cf. also Figure 5).

**Figure 3:**
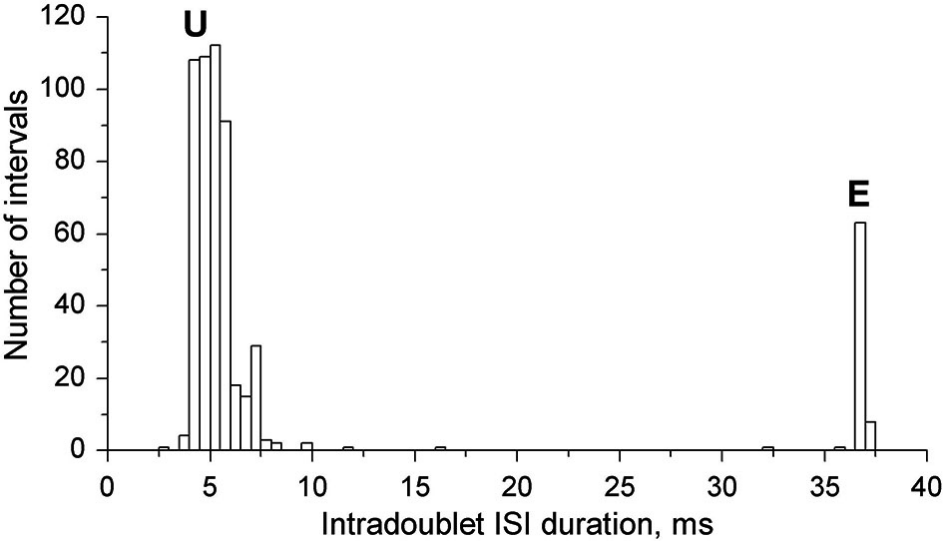
**Intradoublet ISI histogram.** Note some outsiders between usual **(U)** and exceptional **(E)** ISIs.

In Figure 3 the joint intradoublet ISI histogram for SOL MUs is presented with expanded scale. Exceptional doublets (E) create here a narrow maximum at about 37 ms, which differs considerably from the broader maximum of usual doublets (U).

**Table 1:** **Firing characteristics of MU groups**.

**MU group**	**Number of MUs**	**Total number of MU spikes**	**Doublets**			**Triplets**		
			Number	Incidence* [%]	Interval (mean ±SD) [ms]	Number	Incidence* [%]	Interval** (mean ±SD) [ms]
**S**, with single doublets only	12	49231	46	0.09	7.27±2.48			
**R**, with single and repetitive doublets	2	7193	445	6.19	5.05±0.64	6	0.08	36.28±0.22
**E**, with exceptional doublets	2	17462	186	1.07	36.88±0.38			

* calculated with respect to total number of MU spikes

** interval between second and third discharge

### Unusual multiple discharges

**Figure 4:**
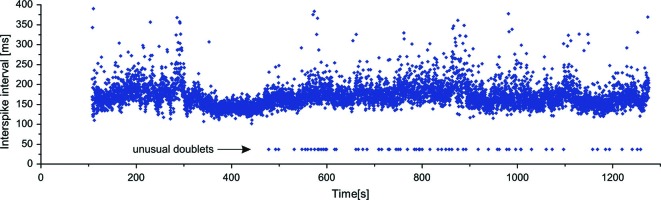
**Long sequence of consecutive discharges of a MU with exceptional doublets**.

As already mentioned above, in two MUs of one subject exceptional doublets were observed, whose intradoublet ISI considerably exceeded the usual limits of 2.5–20 ms (set by the standards of electrophysiological terminology (AAEE, 1987; AAEM, 2001). Figure 4 illustrates a long section of the discharge of a MU with exceptional doublets. The unit was discharging around 6.5 Hz and occasionally slowed down below 5 Hz. It began to fire doublets about 8 min after the start of the experiment and continued until its end.

The intervals of these doublets exhibited much less variability (coefficient of variation 1.04%) than those of the usual doublets (12.0% for repetitive and 21.4% for single doublets). They were also accompanied by the prolonged post-doublet ISI.

**Figure 5:**
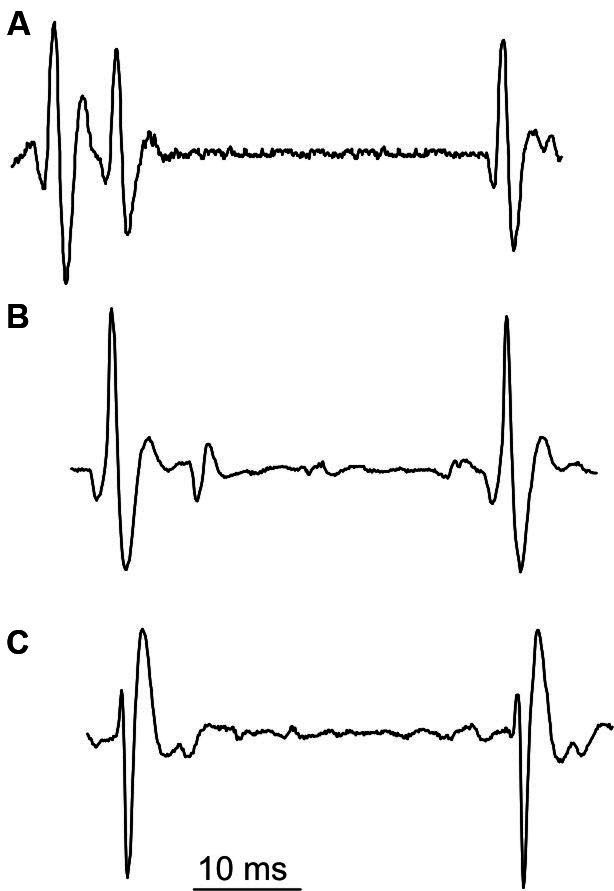
**Unusual discharges. (A)** triplet, **(B)** and **(C)** exceptional doublets (first potential aligned with the second potential in **(A)**).

The special class of unusual multiple discharges are triplets, which are much more seldom than doublets (cf. Table 1). In our experimental data collected from SOL, we encountered only one MU firing triplets. Triplets presented the stereotyped firing pattern (Figure 5): the interval between second and third discharge (triplet ISI) was substantially longer than that between first and second discharge (intradoublet ISI).

Surprisingly, the triplet ISI duration was virtually equal that of the exceptional intradoublet ISI (Figure 5, cf. also Figure 2 and Table 1). This coincidence is quite remarkable given that the exceptional doublets and the triplets were recorded from two different subjects.

## Discussion

This paper presents the doublets recorded from SOL muscle, which were found unexpectedly among single MU data collected for other purposes. SOL is the muscle perhaps most frequently investigated in human studies (e.g., Person and Kudina, 1972; Ashby and Labelle, 1977; Sabbahi and Sedgwick, 1987; Kudina, 1988; Kudina and Pantseva, 1988; Miles et al., 1989; Türker and Miles, 1991; Person and Kozhina, 1992; Kiehn and Eken, 1997; Türker et al., 1997). However, doublets have never been reported in this muscle. Even in the experiments testing the excitability of human MUs within the ISI none of the 141 SOL MUs was found to reveal any sign of increased excitability in the initial interval fragment (Sabbahi and Sedgwick, 1987; Kudina, 1988) in contrast to MUs from flexor carpi ulnaris capable of firing doublets (Kudina and Churikova, 1990). Thus, the occurrence of doublets in SOL must be a very rare phenomenon and the incidence of MNs firing doublets calculated in this study as 9.5%, is by no means severely overestimated.

The question which arises from these data is: why doublets were observed in SOL in these two series of experiments? Firstly, these observations were made in experiments of long duration (approximately 1–2.5 h) and never encountered at the beginning of the experiment (see Figure 5). This seems to have something in common with “warm-up” phenomenon, i.e., the decrease in the MU firing threshold during repeated or sustained contractions (e.g., Gorassini et al., 2002). This phenomenon has been shown to occur in MNs and was attributed to the facilitation of a voltage-dependent persistent inward current (Svirskis and Hounsgaard, 1997; Bennett et al., 1998). Recently, similar mechanism was proposed as the explanation for repetitive doublet firing (Kudina and Andreeva, 2010). It may be hypothesized that such a “warm-up” is necessary also for initiation of single doublet generation. In many earlier SOL studies such long sequences might not have been recorded. Moreover, the doublets might often remain unnoticed, if the researcher was analyzing data with another purpose, and/or was not familiar with their specific firing pattern. It is also important to note that the second spike of the doublet usually has different amplitude and/or shape. Recent studies often rely on the automatic recognition software, which may not mark this discharge as belonging to the same MU.

In official EMG terminology (AAEM, 2001), two potentials of the same MU are classified as a doublet, if the interval between them is shorter than 20 ms. However, in this study we report two cases of MUs with intradoublet ISI above 30 ms. The very low variability of these intervals is remarkable, and even more so is their similarity to the triplet ISI, given that they were recorded from two different subjects.

These unusual phenomena are much more seldom than doublets in SOL *per se*, so there is little chance on their detailed investigation in future. We can only propose the explanation for the triplet interval (below), which is highly speculative and should be treated with caution.

It is commonly accepted that doublets recorded in healthy human muscles are generated in MNs exhibiting delayed depolarization (Kudina, 1974; Partanen, 1979; Bawa and Calancie, 1983; Kudina and Churikova, 1990; Kudina and Alexeeva, 1992a; Garland and Griffin, 1999; Piotrkiewicz et al., 2008; Kudina and Andreeva, 2010; Stephenson and Maluf, 2010), which has a shape of a prominent hump that may spontaneously cross the firing threshold, evoking an extra spike (Granit et al., 1963; Kernell et al., 1964; Calvin, 1974). It is possible that certain MNs possess the second hump situated further down the interspike voltage trajectory. This type of hump would be responsible for the third discharge in triplets and for the exceptional doublets, which are generated by MNs without initial hump responsible for usual doublets. The late hump should be very sharp to explain the extremely low variability of triplet and exceptional intradoublet ISIs.

## Conflict of interest statement

The authors declare that the research was conducted in the absence of any commercial or financial relationships that could be construed as a potential conflict of interest.
